# Patterns and Implications of Gene Gain and Loss in the Evolution of *Prochlorococcus*

**DOI:** 10.1371/journal.pgen.0030231

**Published:** 2007-12-21

**Authors:** Gregory C Kettler, Adam C Martiny, Katherine Huang, Jeremy Zucker, Maureen L Coleman, Sebastien Rodrigue, Feng Chen, Alla Lapidus, Steven Ferriera, Justin Johnson, Claudia Steglich, George M Church, Paul Richardson, Sallie W Chisholm

**Affiliations:** 1 Department of Biology, Massachusetts Institute of Technology, Cambridge, Massachusetts, United States of America; 2 Department of Civil and Environmental Engineering, Massachusetts Institute of Technology, Cambridge, Massachusetts, United States of America; 3 Department of Genetics, Harvard Medical School, Boston, Massachusetts, United States of America; 4 Joint Genome Institute, United States Department of Energy, Walnut Creek, California, United States of America; 5 J. Craig Venter Institute, Rockville, Maryland, United States of America; 6 Department of Biology II/Experimental Bioinformatics, University Freiburg, Freiburg, Germany; University of Toronto, Canada

## Abstract

*Prochlorococcus* is a marine cyanobacterium that numerically dominates the mid-latitude oceans and is the smallest known oxygenic phototroph. Numerous isolates from diverse areas of the world's oceans have been studied and shown to be physiologically and genetically distinct. All isolates described thus far can be assigned to either a tightly clustered high-light (HL)-adapted clade, or a more divergent low-light (LL)-adapted group. The 16S rRNA sequences of the entire *Prochlorococcus* group differ by at most 3%, and the four initially published genomes revealed patterns of genetic differentiation that help explain physiological differences among the isolates. Here we describe the genomes of eight newly sequenced isolates and combine them with the first four genomes for a comprehensive analysis of the core (shared by all isolates) and flexible genes of the *Prochlorococcus* group, and the patterns of loss and gain of the flexible genes over the course of evolution. There are 1,273 genes that represent the core shared by all 12 genomes. They are apparently sufficient, according to metabolic reconstruction, to encode a functional cell. We describe a phylogeny for all 12 isolates by subjecting their complete proteomes to three different phylogenetic analyses. For each non-core gene, we used a maximum parsimony method to estimate which ancestor likely first acquired or lost each gene. Many of the genetic differences among isolates, especially for genes involved in outer membrane synthesis and nutrient transport, are found within the same clade. Nevertheless, we identified some genes defining HL and LL ecotypes, and clades within these broad ecotypes, helping to demonstrate the basis of HL and LL adaptations in *Prochlorococcus*. Furthermore, our estimates of gene gain events allow us to identify highly variable genomic islands that are not apparent through simple pairwise comparisons. These results emphasize the functional roles, especially those connected to outer membrane synthesis and transport that dominate the flexible genome and set it apart from the core. Besides identifying islands and demonstrating their role throughout the history of *Prochlorococcus*, reconstruction of past gene gains and losses shows that much of the variability exists at the “leaves of the tree,” between the most closely related strains. Finally, the identification of core and flexible genes from this 12-genome comparison is largely consistent with the relative frequency of *Prochlorococcus* genes found in global ocean metagenomic databases, further closing the gap between our understanding of these organisms in the lab and the wild.

## Author Summary

*Prochlorococcus*—the most abundant photosynthetic microbe living in the vast, nutrient-poor areas of the ocean—is a major contributor to the global carbon cycle. *Prochlorococcus* is composed of closely related, physiologically distinct lineages whose differences enable the group as a whole to proliferate over a broad range of environmental conditions. We compare the genomes of 12 strains of *Prochlorococcus* representing its major lineages in order to identify genetic differences affecting the ecology of different lineages and their evolutionary origin. First, we identify the core genome: the 1,273 genes shared among all strains. This core set of genes encodes the essentials of a functional cell, enabling it to make living matter out of sunlight and carbon dioxide. We then create a genomic tree that maps the gain and loss of non-core genes in individual strains, showing that a striking number of genes are gained or lost even among the most closely related strains. We find that lost and gained genes commonly cluster in highly variable regions called genomic islands. The level of diversity among the non-core genes, and the number of new genes added with each new genome sequenced, suggest far more diversity to be discovered.

## Introduction

The oceans play a key role in global nutrient cycling and climate regulation. The unicellular cyanobacterium *Prochlorococcus* is an important contributor to these processes, as it accounts for a significant fraction of primary productivity in low- to mid-latitude oceans [1]. *Prochlorococcus* and its close relative, *Synechococcus* [2], are distinguished by their photosynthetic machinery: *Prochlorococcus* uses chlorophyll-binding proteins instead of phycobilisomes for light harvesting and divinyl instead of monovinyl chlorophyll pigments. Although *Prochlorococcus* and *Synechocococcus* coexist throughout much of the world's oceans, *Synechococcus* extends into more polar regions and is more abundant in nutrient-rich waters, while *Prochlorococcus* dominates relatively warm, oligotrophic regions and can be found at greater depths [3]. The *Prochlorococcus* group consists of two major ecotypes, high-light (HL)-adapted and low-light (LL)-adapted, that are genetically and physiologically distinct [4] and are distributed differently in the water column [5,6]. Given their relatively simple metabolism, well-characterized marine environment, and global abundance, these marine cyanobacteria represent an excellent system for understanding how genetic differences translate to physiological and ecological variation in natural populations.

The first marine cyanobacterial genome sequences suggested progressive genome decay from *Synechococcus* to LL *Prochlorococcus* to HL *Prochlorococcus*, characterized by a reduction in genome size (from 2.4 to 1.7 Mb) and a drop in G + C content from ∼59% to ∼30% [7–9]. Notably, genes involved in light acclimation and nutrient assimilation appeared to have been sequentially lost, consistent with the niche differentiation observed for these three groups [7]. This comparison suggested that the major clades of marine cyanobacteria differentiated in a stepwise fashion, leading to patterns of gene content that corresponded to the isolates' 16S rRNA phylogeny.

Recently, however, molecular sequence data and physiology studies have revealed complexity beyond the HL/LL paradigms. Within the LL ecotype, for instance, some but not all isolates can use nitrite as a sole nitrogen source [10], and the LL genomes range widely in size [7,8]. Moreover, the distribution of phosphate acquisition genes among *Prochlorococcus* genomes does not correlate to their rRNA phylogeny but instead appears related to phosphate availability: strains isolated from low-phosphate environments are genetically better equipped to deal with phosphate limitation than those from high-phosphate environments, regardless of their 16S rRNA phylogeny [11]. Thus, while the HL/LL distinction has held up both phenotypically and genotypically, there are other differences among isolates that are not consistent with their rRNA phylogeny. Thus, to understand diversification and adaptation in this globally important group, we must characterize the underlying patterns of genome-wide diversity.

Lateral gene transfer (LGT) is one mechanism that creates complex gene distributions and phylogenies incongruent with the rRNA tree. The question of whether a robust organismal phylogeny can be inferred despite extensive LGT is still hotly debated [12,13]. If a core set of genes exists that is resistant to LGT, then gene trees based on these core genes should reflect cell division and vertical descent, as has been argued for the gamma *Proteobacteria* [13]. Others argue that genes in a shared taxon core do not necessarily have the same evolutionary histories, making inference of an organismal phylogeny difficult [14]. In spite of this debate, the core genome remains a useful concept for understanding biological similarity within a taxonomic group. Recent comparisons within the lactic acid bacteria, cyanobacteria, and Streptococcus agalacticae groups, for instance, have each revealed a core set of genes shared by all members of the group, on top of which is layered the flexible genome [15–17]. The vast majority of genes in the core genome encode housekeeping functions, while genes in the flexible genome reflect adaptation to specific environments [16] and are often acquired by LGT. Thus the core and flexible genomes are informative not only in a phylogenetic context, for understanding the mechanisms and tempo of genome evolution, but also in an ecological context, for understanding the selective pressures experienced in different environments.

To further understand diversification and adaptation in *Prochlorococcus*, we obtained sequences of eight additional genomes representing diverse lineages, both LL- and HL-adapted, spanning the complete 16S rRNA diversity (97% to 99.93% similarity) of cultured representatives of this group [18–22] (Table 1). Comparing these genomes with available genomes for *Prochlorococcus* and marine *Synechococcus*, our goal was to reconstruct the history of vertical transmission, gene acquisition, and gene loss for these marine cyanobacteria. In particular we identified functions associated with the core and flexible genomes and analyzed the metabolic pathways encoded in each. This analysis reveals not only what differentiates *Synechococcus* from LL *Prochlorococcus* from HL *Prochlorococcus*, but also informs our understanding of how adaptation occurs in the oceans along gradients of light, nutrients, and other environmental factors, providing essential biological context for interpreting rapidly expanding metagenomic datasets.

**Table 1:**
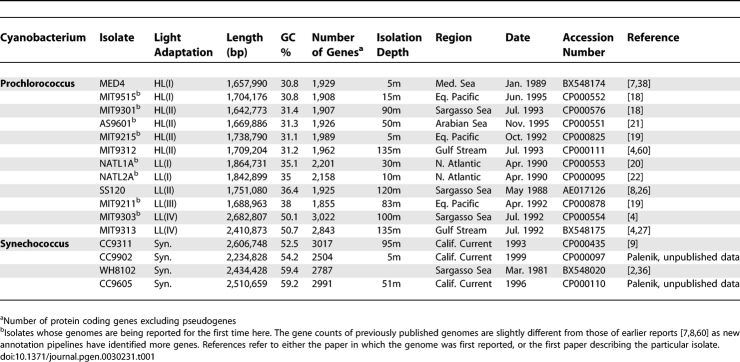
General Characteristics of the *Prochlorococcus* and *Synechococcus* Isolates Used in This Study

## Results/Discussion

### Core Genome

**Figure 1:**
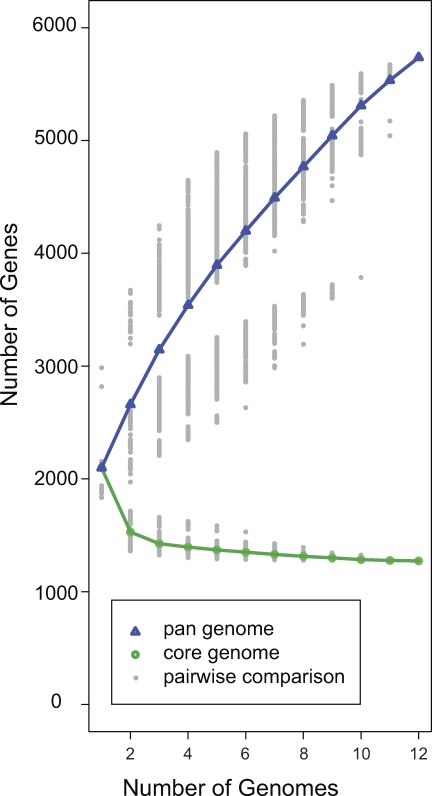
The Sizes of the Core and Pan-Genomes of *Prochlorococcus* The calculated sizes depend on the number of genomes used in the analysis. If k genomes are selected from 12, there are 12!/(k!(12 − k)!) possible selections from which to calculate the core and pan-genomes. Each possible selection is plotted as a grey point, and the line is drawn through the average. This analysis is based on a similar one in [15].

The genomes of 12 *Prochlorococcus* isolates, representing all known major phylogenetic clades, range in size from 1.6 Mbp (MIT9301) to 2.7 Mbp (MIT9303) (Table 1). As more genomes are compared, we observe an asymptotic decline in the number of shared (core) genes (Figure 1), similar to observations for *Streptococcus* genomes [15]. This suggests a finite size of the core genome of approximately 1,250 genes, or 40% to 67% of the genes of any particular isolate. In contrast, the pan-genome [15,23] of these isolates, encompassing the core genes, plus the total of all additional genes found in any of the isolates (the “flexible genes”), contains 5,736 genes (Table S1). The gene accumulation curve as more genomes are added to the analysis is clearly far from saturated (Figure 1), indicating a far larger gene pool within the *Prochlorococcus* clade than is captured by our sequenced isolates, and suggesting the presence of *Prochlorococcus* lineages in the wild, with yet-to-be discovered traits.

Although the closely related marine cyanobacterium *Synechococcus* commonly coexists with *Prochlorococcus*, it is considered more of a generalist, and, collectively, is capable of growth over a broader range of nutrient concentrations and temperatures than is *Prochlorococcus*. To understand the divergence of marine *Synechococcus* and *Prochlorococcus* since their last common ancestor, we looked for genes present in all *Prochlorococcus* but absent from some or all *Synechococcus*. We found 33 such genes, 13 of which are not found in any sequenced marine *Synechococcus* (Table S2). Eight of these *Prochlorococcus*-only genes have been assigned putative functions including one HL inducible protein (MED4′s *hli11*, which responds only slightly to light stress [24]), a possible sodium-solute symporter, an iron-sulfur protein, and a *deoR*-like transcription factor, but it is unclear what role these genes have in distinguishing *Prochlorococcus* from *Synechococcus*. Perhaps more importantly, the differentiation between these two groups is defined by the absence in *Prochlorococcus* of 140 genes that are present in all four sequenced marine *Synechococcus* (Table S3). All *Prochlorococcus* isolates sequenced to date lack, for example, divinyl protochlorophyllide a reductase (*dvr*) [25], resulting in one of the defining phenotypic properties of *Prochlorococcus*: divinyl chlorophyll *a* as the primary light harvesting pigment [26]. Other light harvesting genes absent in *Prochlorococcus* include allophycocyanin (*apcABCDE*), some phycoerythrins, and phycobilisome linkers. *Synechococcus* also possess several molybdopterin biosynthesis enzymes not found in *Prochlorococcus* (*mobA*, *moaABCDE*), which may be necessary for the function of nitrate reductase [27,28]. Although all 12 *Prochlorococcus* isolates also lack the gene for nitrate reductase, this might be a result of the isolation conditions, and further study may reveal nitrate-utilizing isolates [29].

The underpinnings of *Prochlorococcus* diversity should be reflected in the respective roles of the core and flexible genomes. If the core genome provides for central metabolic needs shared by all isolates, it should be possible to reconstruct those pathways with the core genes alone. Therefore we asked whether the core genome encodes all the biochemical pathways needed for growth from the nutrients available to *Prochlorococcus* using Pathway Tools [30] and compared the resulting map with the manually curated, but less detailed, metabolic map for *Prochlorococcus* SS120 [8]. The automated approach is more detailed (Figures S1–S4 and see http://procyc.mit.edu), but the results recapitulate the previous manual effort.

We have identified core genes responsible for nearly all the reactions in the central metabolism, from the Calvin Cycle to the incomplete TCA cycle, including pathways to synthesize all 20 amino acids, several cofactors, and chlorophylls (Figures S1–S4). Among the genes that were assigned functions in the *Prochlorococcus* SS120 core metabolic model, all but seven are found to be part of the core genome in this study. Five of these seven additional genes in SS120 are transporters: SS120_12271, an iron or manganese transporter; SS120_15671, a sodum/alanine symporter; and SS120_06831–06851, three genes encoding an ABC-type amino acid transporter. The other two, *sdhA* and *sdhB*, are putatively responsible for the conversion of fumarate to succinate in the incomplete TCA cycle, but they have no apparent orthologs in many *Prochlorococcus* isolates. Importantly, *sdhAB* in the TCA cycle and *pdxH* in pyridoxal phosphate synthesis are the only cases in which one of the pathways examined could be reconstructed in some strains, but not in the core genome. An additional case, the phosphorylation of pantothenate in coenzyme A synthesis, is incomplete in the core and pan reconstructions, indicating that we have most likely failed to identify the gene or an alternate pathway (Figure S4). This observation supports the view in which essential life functions are unchanging across all *Prochlorococcus*, while nonessential or environment-specific functions are found in the flexible genome (see below). The functions of the latter, then, may relate to niche-specific adaptations that are not required for growth under optimal conditions, but that provide a fitness advantage in particular habitats. The pattern of their gain and loss in phylogenetic space could therefore help us understand when and how *Prochlorococcus* lineages evolved adaptations to particular environments. However, a close examination of their gain and loss requires a robust phylogenetic tree as a scaffold for analysis.

### Phylogeny of *Prochlorococcus* Isolates Using the Core Genomes

**Figure 2:**
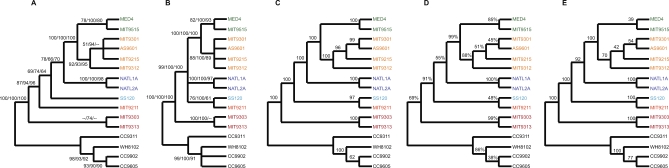
Phylogenetic Relationship of *Prochlorococcus* and *Synechococcus* Reconstructed by Multiple Methods (A) 16S rRNA and (B) 16S-23S rRNA ITS region reconstructed with maximum parsimony, neighbor-joining, and maximum likelihood. Numbers represent bootstrap values (100 resamplings). (C) Maximum parsimony reconstruction of random concatenation of 100 protein sequences sampled from core genome. Values represent average bootstrap values (100 resamplings) from 100 random concatenation runs. (D) Consensus tree of all core genes using maximum parsimony on protein sequence alignments. Values represent fraction of genes supporting each node. (E) Genome phylogeny based on gene content using the approach of [34]. Values represent bootstrap values from 100 resamplings.

Identification of the core genome shared by all *Prochlorococcus* isolates provides a new opportunity for determining the phylogenetic relationship among isolates. Our current understanding of the branching order among isolates is based on single gene phylogenies including 16S rRNA [10], 16S-23S rRNA internal transcribed spacer sequence (ITS) [18], *rpoC1* [31], *psbA* [32], and *petBD* [6]. Although trees based on these genes generally agree on the phylogenetic position of most isolates, they disagree, or lack bootstrap support, for the branching order of internal nodes among LL isolates (see Figure 2A and 2B for 16S rRNA and ITS trees). To reconstruct a robust phylogeny, we randomly concatenated 100 protein sequences from a pool of all core genes and compared the topology of the resulting trees (Figure 2C), analogous to the approach described by Rokas and co-workers [33]. This random concatenation was repeated 100 times and the same highly supported topology emerged every time. This tree is very similar to the 16S rRNA tree (Figure 2A) except for the position of LL isolates MIT9211 and SS120. We attribute this discrepancy to the limited information in any single gene (including 16S rRNA), and our analysis suggests that MIT9211 and SS120 form a separate clade. Each node in the concatenated protein tree is also supported by a plurality of individual core genes (as defined above) (Figure 2D). Based on these results, we postulate that this tree represents the most probable evolutionary relationship among *Prochlorococcus* isolates. However, it is unclear if the physiology of SS120 and MIT9211 warrants considering them as one or separate ecotypes. Furthermore, many single gene phylogenies supported alternative topologies for this node, and future analyses with more genomes or alternative phylogenetic approaches may result in different topologies for this node.

The history of *Prochlorococcus* is marked not only by sequence divergence among the core genes, but also by the gain and loss of genes. We constructed a dendrogram based on the presence or absence of individual orthologous groups (Figure 2E) [34]. Again, the topology of this tree is identical to that of Figure 2C. This suggests that shared gene content among *Prochlorococcus* isolates is significantly influenced by the isolates' phylogenetic relationship despite the occurrence of lateral gene gain and loss.

### Flexible Genome

#### Patterns of gene gain and loss in the evolutionary tree.

**Figure 3:**
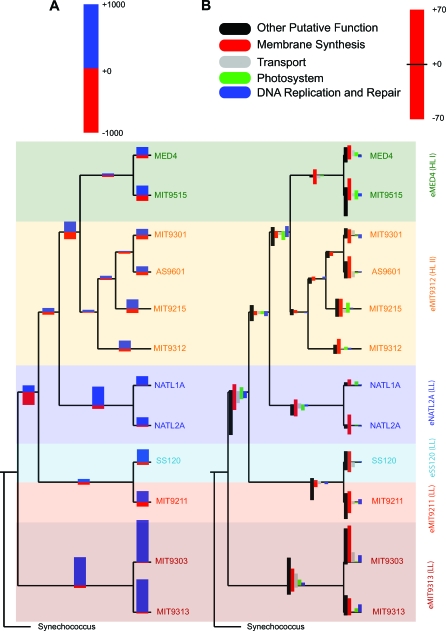
The Loss and Gain of Genes through the Evolution of *Prochlorococcus* The ancestor node in which a gain or loss event took place was estimated by maximum parsimony. Four marine *Synechococcus* genomes (not shown) were included in the calculation, and the phylogenetic tree from Figure 2C was rooted between the *Synechococcus* and *Prochlorococcus* lineages. (A) The total number of genes gained and lost at each node. (B) The loss and gain of genes in that could be assigned functional roles through homology. Note that (B) focuses on the small minority of genes that do have an assigned function. Genes were assigned to one of five categories on the basis of keyword matches against the gene name or COG description. “Other Putative Function” refers to genes with assigned function but not belonging to the four major categories. Note the difference in scale for (A and B).

We used our most probable phylogenetic tree (Figure 2C) as a map for the evolution of each isolate and superimposed the gain and loss of flexible genes (i.e., non-core) upon it (Figure 3A). By assigning costs to gain and loss events (see Methods) and then minimizing the total cost (maximum parsimony criterion), we estimated for each gene in each node of the tree whether it was more likely to have been inherited from a common ancestor or acquired at that node [35].

As mentioned above, 140 genes found in all *Synechococcus* are absent in all *Prochlorococcus* (Table S3). This is consistent with our earlier image, based on only four genomes, of progressive gene loss from *Synechococcus* to LL *Prochlorococcus* to HL *Prochlorococcus* [7,8,36]. However, our analysis suggests an alternative to this view, in that the MIT9313 lineage (i.e., the MIT9313/MIT9303 “cluster” or eMIT9313 clade, *sensu* [37]) is not simply an intermediate step in this gene loss process. Although the genome sizes within eMIT9313 are similar to those of *Synechococcus*, the eMIT9313 clade appears to have gained a large number of genes, including many unique to each isolate. These genes are not found in any other sequenced *Prochlorococcus* or *Synechococcus* strain, and the eMIT9313 strains may therefore have acquired them after their divergence from the other *Prochlorococcus*. The large difference between strains MIT9313 and MIT9303 is then most likely the result of further gene gains after they diverged from each other. After the divergence of eMIT9313, all *Prochlorococcus* genomes have a roughly constant size (1.66 to 1.84 Mbp). However, we still observe significant gene gain and loss. A few particular examples are discussed below, but additional work remains to show how these dynamics contribute to the distribution patterns we observe in the oceans for specific lineages.

#### Ecotypic differences: Genes underlying the HL/LL ecotypes.

As described in many previous studies, *Prochlorococcus* can be classified into two broad groups based on their growth adaptation to specific light intensity (and corresponding phylogeny) [4]. In addition to the core genome shared by all 12 *Prochlorococcus* examined in this study, HL isolates all share an additional 257 genes, 95 of which are not found in any of the LL isolates (Table S6). This HL core provides further clues to the genetic bases for the HL/LL physiological and ecological differentiation that has been observed in previous works [4,5,19,20,37–42]. All HL isolates carry an operon containing a DNA ligase, exonuclease, and helicase, which might be involved in DNA repair or other nucleic acid processing. HL isolates also possess large numbers of HLIPs (although NATL1A and NATL2A have more), which are thought to protect photosystems from oxidative damage [39] and are upregulated in stress conditions such as high light [24], nitrogen starvation [43], and phage infection [44]. In particular, they share at least three additional genes for HL inducible proteins not found in any other strain. In addition to HL stress, one (*hli8/18* in MED4) is upregulated in response to phage infection, and the other two (*hli15* and *hli22*) by nitrogen starvation [43,44]. The HL isolates also share some genes with no clear connection to photobiology, such as a uridine kinase that may provide an alternative pathway for uracil recycling to UMP. In all *Prochlorococcus*, UMP can be generated by core pathways involving the core *upp* or *pyrBCDEF* genes [45]. All HL isolates also share the operon *tenA-thiD*, which may be involved in thiamine salvage and/or degradation [46,47]. In addition, the HL core contains dozens of hypothetical and conserved hypothetical genes not found in any LL isolate, and these might be critical for survival in the commonly nutrient-poor, HL environment of the surface oceans. Finally, all HL and eNATL2A isolates (which are LL, but closest to the HL clade) include at least one photolyase (orthologs of P9301_3091) and a second possible (P9301_03091), and some HL strains have a third (P9301_03921), the function of which is to repair UV-induced DNA lesions (Table 2).

**Table 2:**
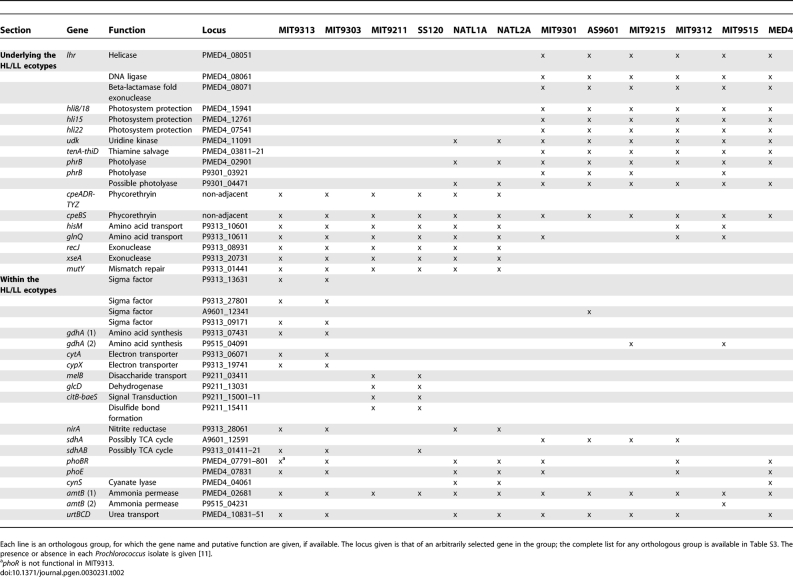
Non-core Genes Referred to in the Discussion

Likewise, LL isolates share an additional 92 genes beyond the *Prochlorococcus* core, 48 of which are not found in any HL isolates (Table S7). All *Prochlorococcus* have lost the majority of genes involved in phycobilisome synthesis but LL isolates retain several phycoerythrin genes (*cpeABSTYZ*), whereas HL isolates have lost all but *cpeB* and *cpeS*, consistent with previous observations based on fewer genomes [48]. The role of phycoerythrin in *Prochlorococcus* remains uncertain, but may be related to signal transduction rather than light harvesting [49,50]. Individual *Prochlorococcus* strains possess different complements of amino acid transporters. But all LL isolates, and only some HL isolates, contain the tandemly arranged amino acid transporter components *glnQ* and *hisM*, suggesting some variation among *Prochlorococcus* ecotypes in the ability to take up amino acids [51].

Several exonucleases that repair UV-induced lesions, encoded by *recJ* and *xseA*, are exclusive to LL isolates, which is surprising given their reduced exposure to UV radiation. These genes might be necessary to protect against UV exposure during mixing events, and their absence from HL isolates suggests the HL isolates have different strategies to limit DNA damage. Moreover, LL isolates exclusively encode *mutY*, whose product prevents mutations arising from oxidatively damaged guanine residues [52]. The absence of the *mutY* gene in HL *Prochlorococcus* has been hypothesized to underlie their extremely low %G + C content, by increasing the frequency of G-C to A-T mutations [7]. However, this gene is present in LL isolates with %G + C as low as 35%, suggesting that *mutY* alone is not responsible for genomic A + T enrichment [53].

#### Ecotypic differences: Clades within the HL and LL ecotypes.

Going beyond the HL and LL ecotypes, two distinct subclades have been identified within the HL ecotype (eMED4 and eMIT9312), and several lineages within the LL ecotype (eNATL2A, eMIT9313, and eSS120 + eMIT9211) [18] (Figure 3). The distribution of cells belonging to these subclades has been measured along extensive environmental gradients in the oceans, and the two HL subclades have distinct distributions most strongly correlated with surface temperature [39,40]. Moreover, two LL clades (eNATL2A and eMIT9313) have distinct distributions as well: cells related to eNATL2A can be abundant at the surface, while cells related to eMIT9313 are generally found at the base of the euphotic zone in stratified waters and never at the surface [40]. This is in spite of the two clades' similar optimum light intensity for growth [19,40]. Given these ecological distinctions, we looked for genes distinguishing these subclades (Table 2).

The eMIT9313 clade has many features that distinguish it from other *Prochlorococcus* (Table S8). Acquired genes include multiple sigma factors and kinases, likely involved in signal transduction, outer membrane synthesis enzymes, and transporters. Their possession of transporters not found in other *Prochlorococcus* or in *Synechococcus* may imply that they are exploiting nutrient resources unique to their environment, or they may simply have experienced weaker selection for reducing genome size. Likewise, the two isolates in this clade (MIT9313 and MIT9303) share three sigma factors (MIT9303 has a fourth) and several other transcriptional regulators not found in any other isolate, suggesting they have more complexity in their ability to respond to various stimuli. The eMIT9313 isolates also share a glutamate dehydrogenase gene (*gdhA*), absent from most other *Prochlorococcus* (two HL isolates share a distantly related allele), which provides an alternative pathway for ammonium incorporation besides the standard GS-GOGAT pathway. This enzyme has been shown in *Synechocystis* to be important during the late stages of growth when energy is limiting, and for ammonia detoxification [54]. We also observe that photosystem II genes *psbU* and *psbV* are exclusively found in eMIT9313 (as well as most other cyanobacteria) along with possible electron transporters (*cytA*, *cypX*). The eMIT9313 isolates carry only three *pcb* genes, encoding light harvesting antenna proteins, compared to six or seven in the other LL isolates. This relative lack of *pcb* genes, however, does not seem to prevent growth at very low irradiances, as eMIT9313 cells are often found at the base of the euphotic zone. The eMIT9313 isolates also have relatively few genes for HLIPs (nine in eMIT9313, compared to 12–13 in SS120/MIT9211 and 41 in eNATL2A), which might help explain why this clade is not found in surface waters.

Five genes with assigned functions were unique to eSS120/eMIT9211 (P9211_03411, P9211_13031, P9211_15001, P9211_15011, P9211_15411), but there were no clear linkages between these genes and the distribution pattern of this group in the ocean.

In contrast, the eNATL2A isolates (NATL1A and NATL2A), whose low optimum light intensity for growth marks them as LL [19,40] have some notable HL-like properties. The eNATL2A isolates possess photolyase genes, like HL isolates, and they harbor more genes for HLIPs than any other HL or LL isolate. Together these genes may help explain the abundance of eNATL2A at the surface relative to other LL clades [40]. They also share the uridine kinase found in HL isolates.

All isolates in the eMIT9313 and eNATL2A clades possess a nitrite reductase gene, *nirA*, whereas no other *Prochlorococcus* lineages (HL or LL) have this gene, a difference that has been confirmed through physiology studies [10]. The availability of nitrite may therefore influence the distribution of these two clades, although this pattern has not emerged in the field studies to date [39,41].

In spite of their different distributions in the ocean, we could identify only one gene with a described function that distinguishes the two HL clades eMIT9312 and eMED4. All isolates in eMIT9312 possess a gene similar to *sdhA* which encodes succinate dehydrogenase. Unlike the proteobacteria-like *sdhA* found in SS120, MIT9313, and MIT9303 and previously assigned to the incomplete TCA cycle [8], the HL gene is actinobacteria-like and is not accompanied by *sdhB*, raising the possibility that this dehydrogenase/reductase acts on a different substrate. Temperature variability is most strongly correlated with differences in the abundances of eMED4 and eMIT9312 along a longitudinal gradient in the oceans, and this is consistent with the temperature limits for growth for strains representing these ecotypes in culture [39]. These properties could emerge from differences within orthologous proteins, yielding different enzymatic reaction temperature optima, rather than from the presence or absence of entire genes. This complicates the search for ecotype-defining genes in their case.

#### Isolate-specific genes.

We found that a large fraction of variability was in the “leaves of the tree,” that is, genes gained by one isolate but not necessarily by others in the same clade (Figure 3B and Table S4). The greatest differentiator between the most closely related isolates are genes related to outer membrane synthesis (Table S5). For example, while MIT9515 and MED4 each have several genes in COG438 and COG451 (both COGs described as acyltransferases connected to outer membrane synthesis), these genes are only distantly related [55]. Six genes matching COG438 are found in MIT9515 but not MED4, and these six all have best matches to genes in lineages outside *Prochlorococcus*. The rapid turnover of genomic content contrasts with the broader similarity of their roles: even though the genes found in different isolates are not orthologs and have little to no sequence similarity, they share the same biological role. Such membrane synthesis genes were probably lost or gained continuously throughout the evolution of *Prochlorococcus*, as every ancestor node is estimated to have lost or gained some in that category (Figure 3B).

Certain cell surface proteins are potentially under strongly diversifying selection if they serve as attachment or recognition sites for predators or phages. The observed variation among genomes in relation to this category supports this idea and suggests that the predatory environment could be different in each of the locations where these isolates originated. However, it is deceptive to consider these the most recent changes, as there are innumerable undiscovered *Prochlorococcus* genotypes in the wild, some of which could fill the gap between MIT9515 and MED4, for example. Such variation, some of which may be adaptive, is below the resolution of current methods for measuring ecotype abundance in the oceans [39,42,56].

After cell surface synthesis, the next largest fraction of the flexible genome is transporters (Figure 3B). As discussed above, the larger genomes of MIT9303 and MIT9313 have a significant number of transporters not shared with other *Prochlorococcus*, although some are shared with *Synechococcus*. Among their predicted substrates are toxins, sugars, and metal ions. Relatively few transporters are specific to the other LL isolates. In addition, each HL isolate possesses a different set of transporters, but there is no set both universal among HL isolates and absent from LL isolates. Furthermore, the presence of specific transporters does not follow the phylogeny of the HL ecotype. Transport genes must therefore be subject to rapid gain and loss, such that their presence is not conserved within the subclades. Transport reactions are peripheral to metabolic pathways, and such peripheral reactions are predicted to be subject to the most rapid turnover [57].

Individual *Prochlorococcus* isolates also contain multiple copies of specific light-related genes but in different numbers. MED4, the first HL genome to be studied, has only one *pcb* light harvesting antenna gene whereas the first LL genomes had two (MIT9313) or eight (SS120) [58]. Our new data identify MED4 as the exception, since the other five HL isolates share a second copy in the same well-conserved neighborhood. Surprisingly, there is huge variation in the number of genes encoding HLIPs, ranging from nine in eMIT9313 to 41 in eNATL2A. Even at the leaves of the tree, within the HL clades, HLIPs range in copy number from 15 to 24.

A second copy of the core photosystem II gene *psbA* also appears in more than half the genomes. This gene is especially interesting because it is also found in all *Prochlorococcus*-infecting myoviruses and podoviruses sequenced to date [59]. While it is possible that *psbA* might have been inserted into the genome by those viruses, much as the genes in genomic islands are thought to have been [60], the similarity between *psbA* copies in the same genome suggests they are the result of intragenomic duplication events, not transduction. Indeed, in all of these strains the two copies are identical or nearly identical in nucleotide sequence, suggesting that they result from a very recent duplication event. Furthermore, while extra *psbA* copies sometimes appear in islands, they do not always. In MIT9515, for example, the two copies lie in tandem but not in an island. It is not clear why *psbA* is subject to such duplication events while other photosystem genes are not. The most likely reason is that the PsbA protein (D1) has an exceptionally brief half-life due to light-induced damage [61], and therefore two gene copies help ensure sufficient product via a gene dosage effect and/or by promoter differences leading to expression under different conditions.

The complement of nutrient assimilation genes also varies among the most closely related isolates, suggesting frequent gain and loss events. Such variability was recently described for genes involved in phosphorus assimilation [11]. Within the eMIT9312 clade, for instance, the isolates AS9601 and MIT9215 are lacking the *phoBR* two-component system, the *phoE* porin, and several related genes that are present in MIT9312 and MIT9301. Now equipped with whole genomes for 12 isolates, we see a similar situation for nitrogen assimilation genes. MED4 is the only HL isolate with cyanate lyase, and likewise MIT9515 exclusively carries a second ammonia permease gene. In contrast, MIT9515 is the only HL isolate lacking urea transport and metabolism genes. This variability may reflect the available nitrogen sources in the local environment where these isolates originated, as has been hypothesized for phosphorus [11].

### Chromosomal Location of the Flexible Genome

Previous work comparing the genomes of two closely related *Prochlorococcus* isolates has highlighted the importance of highly variable island regions in genomes as the sites of genomic variation [60]. These variable genome segments appear to contain genes that could be important for adaptation to local conditions, and include many of the functions encoded in the flexible genome analyzed here, such as outer membrane synthesis. Thus, we analyzed the chromosomal geography of the flexible genome. Are flexible genes preferentially located in island regions, and if so are the most recently acquired genes more likely to be island genes?

**Figure 4:**
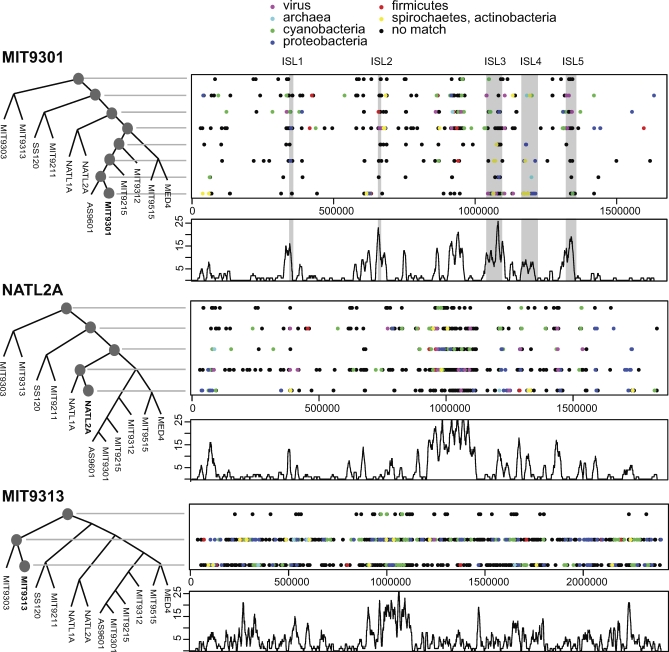
Gene Acquisitions Confirm Known, and Identify Novel, Genomic Islands in *Prochlorococcus* The dot plots indicate the location on the chromosome and the ancestor node in which the gene is estimated to be gained. The color indicates where the best match was found. In MIT9301, The shaded regions are islands as defined by [60]. Gained genes are defined for each node as in Figure 3. The lower plot is the number of genes gained in a sliding window (size 10,000 bp, interval 1,000 bp) along the chromosome.

To answer these questions, we plotted the timing of gene gain events against their chromosome positions (Figures 4 and S5 and S6). In HL isolates, the islands contain the majority of gained genes. Furthermore, the islands include not only recent acquisitions but also genes that were gained long ago, based on their presence in divergent modern isolates. However, particular islands show different levels of gain or loss events throughout the evolution of *Prochlorococcus*. Apparently, these sites have been important for adaptation throughout the history of most *Prochlorococcus* lineages.

In the earlier comparison of two genomes at a time, islands were identified as breaks in syntenic regions [60]. Among LL isolates, this approach is difficult because the genomes are more divergent, and numerous rearrangements have disrupted synteny, even for core genes. Plotting gene gain events along the chromosome, however, reveals island structure in several LL genomes. MIT9211 and SS120 have clearly defined islands much like the HL isolates, while NATL1A and NATL2A have one large potential island and several much smaller sites (Figures 4 and S5 and S6).

Surprisingly, this approach is less helpful in the two large genomes, MIT9313 and MIT9303, which have apparently gained a large number of genes throughout the chromosome (Figure 4). In their organization and content, the large genomes are exceptional among *Prochlorococcus* in three ways: they share a large number of genes with *Synechococcus* that the other isolates do not, they gain additional genes not shared with any other *Prochlorococcus* or with marine *Synechococcus*, and those genes do not cluster in discernible islands. The first two differences mean that their genome sizes are much greater than those of the other isolates. The lack of islands together with the larger genome size could indicate that these isolates have acquired genes through a different mechanism that does not direct them toward islands. The relative lack of pressure towards genome reduction in the evolution of eMIT9313 may also play a role. However, additional sequenced genomes may provide better coverage of the eMIT9313 clade and clarify the timing of gene gain events.

### The Frequency of Core and Flexible Genes in Wild Populations

**Figure 5:**
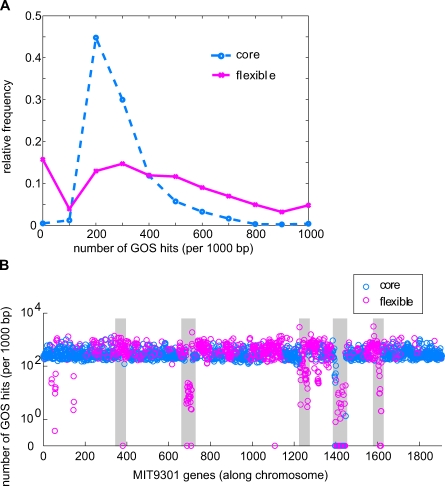
*Prochlorococcus* Core and Flexible Genes in the Global Ocean Survey (GOS) Dataset [64] (A) Frequency distribution of GOS hits per gene, using genes in the *Prochlorococcus* MIT9301 genome as queries. Most core genes retrieve a similar number of GOS hits, as one would expect from single copy genes shared by all *Prochlorococcus*, resulting in a relatively tight frequency distribution. In contrast, flexible genes retrieve a broad range of GOS hits per gene, consistent with their scattered distribution among genomes. (B) The number of GOS hits per gene, again using MIT9301 genes as queries, plotted against position along the chromosome. Shaded regions represent genomic islands, after [60]. Flexible genes with low representation in the GOS dataset tend to be located in genomic islands. In both (A) and (B), the number of GOS hits per gene is normalized to gene length and plotted as hits per gene, per 1,000 bp.

Because *Prochlorococcus* is very abundant in many regions of the oceans that have recently been sampled and subjected to metagenomic analysis [62–64], we have an opportunity to test the robustness of our distinction between core and flexible genes in *Prochlorococcus.* If the core genome we have defined, based on the genomes of 12 isolates, is reasonably universal and core genes are generally single copy per genome, we would expect to find core genes represented with equal frequency in the ocean; the occurrence of non-core genes, in contrast, would be more variable. To test this hypothesis, we used the MIT9301 core and flexible genomes as queries against the Global Ocean Survey dataset [64], as MIT9301 often shares the highest sequence similarity with GOS sequences. As expected, the core genes, after normalization to gene size, are represented in roughly equal abundance in the database, with only a few exceptions (Figure 5A). In the case of non-core, or flexible genes, many had few or no hits, and a few were even more abundant than the average core gene, suggesting more than one copy per genome (Figure 5A). Seven core genes are underrepresented in the GOS dataset relative to other core genes, and all seven are located in a genomic island in MIT9301 largely related to cell surface biosynthesis (Figure 5B). The most abundant flexible genes encode HLIPs and hypothetical proteins and are also found in islands in MIT9301 (Figure 5B). This supports the hypothesis that islands are dynamic reservoirs for recent and local adaptation.

### Conclusion

In this study we have attempted to advance our understanding of the evolutionary origins of diversity in *Prochlorococcus* by defining the core and flexible genomes and examining the patterns of gain and loss of non-core genes over the course of evolution. We have learned, for example, that many genes involved in adaptation to different light intensities and DNA repair were apparently fixed before the modern clades diverged, and as a result, the HL-/LL-adapted dichotomy has persisted both genetically and phenotypically. The eNATL2A clade appears to be a refinement on the HL/LL paradigm, as its isolates grow optimally at light intensities typical of the LL ecotype, but have the photoprotective abilities of the HL ecotype. More recent changes in genome content, i.e., those occurring at the tips of the phylogenetic tree, involve cell surface features that are likely under selection pressure via predators and phage and transporter composition, which likely plays a role in both defense from toxins and differences in nutrient availability. The latter is consistent with our earlier observation that genes involved in phosphorus acquisition are distributed among *Prochlorococcus* isolates not according to phylogeny, but rather the P concentrations in their ocean of origin [11]. However, despite the clear evidence for common gene gains and losses throughout the evolution of *Prochlorococcus*, we still observed a significant correlation between genome content and phylogeny. This suggests an important contribution of vertically inherited genes to the overall genome content that cannot be easily substituted through lateral gene transfer or lost altogether.

The core genome of *Prochlorococcus*, with 81% of the 1,273 genes having an inferred function, is now reasonably well understood and appears to encode a viable cell. That this could be circumscribed through the analysis of only 12 genomes is encouraging, and likely emerges from the reasonably small evolutionary distance between these isolates. The close agreement between manually curated core pathway reconstruction for one isolate [8], and the automatic reconstruction of the core metabolism shared by all 12 isolates in our study, promises to help streamline the analysis of new genomes. To date, discussions of minimal genomes to support life have focused on the set of genes that enable heterotrophic cells to replicate on rich organic media, where they benefit from nutrients that must have been synthesized by other organisms [65]. Here, however, we are approximating the minimum number of genes necessary to convert solar energy, carbon dioxide, and inorganic nutrients to living biomass.

The *Prochlorococcus* flexible genome is still only loosely defined, as over 70% of the orthologous groups in this category have no known homolog in MicrobesOnline and no inferred function. Moreover, as the last genomes are added to the analysis, they each add roughly 150 new genes to the *Prochlorococcus* pan-genome (Figure 1); thus it appears that the global pool of genes that are residing, at this moment, in a *Prochlorococcus* cell cannot even be approximated from this dataset. Therefore, one of the most daunting unanswered questions is: How many *Prochlorococcus* genotypes truly exist in the ocean, and what fraction of these has differential fitness at any point in time?

The level of diversity found in the flexible genes, and the steady increment of genes added to the *Prochlorococcus* pan genome with each new genome, suggests that we have barely begun to observe the extent of micro-diversity among *Prochlorococcus* in the ocean. Although the sequencing of 12 genomes represents one of the larger sequencing projects of closely related isolates to date, each isolate undoubtedly represents a subclade of a very large number of cells—especially considering the approximately 10^25^ *Prochlorococcus* cells in the ocean [3]. Additional sequencing, especially metagenomic [63] and single-cell sequencing [66], will help us understand more about on what scale, and where in the genomes, the flexible genes vary. In particular, it will be enlightening to understand the complete genome diversity of the 10^5^ cells in a milliliter of ocean water, and conversely, how widely separated in space two cells with identical genomes might be.

## Materials and Methods

### DNA sequencing and assembly.

The genome sequences of eight of the isolates used in our analysis are reported for the first time here. The genomes of MIT9211, MIT9515, NATL1A, MIT9303, MIT9301, and AS9601 were sequenced by the J. Craig Venter Institute as follows: Two genomic libraries with insert sizes of 4 and 40 kb were made as described in [67]. The prepared plasmid and fosmid clones were sequenced from both ends to provide paired-end reads at the J. Craig Venter Institute Joint Technology Center on ABI3730XL DNA sequencers (Applied Biosystems). Successful reads for each organism were used as input for the Celera Assembler. WGS sequence produced by the assembler was then annotated using the PGAAP at NCBI. Accession numbers for all genomes are provided in Table 1.

NATL2A was sequenced at the DOE Joint Genome Institute by methods described previously (http://www.jgi.doe.gov/sequencing/protocols/prots_production.html). Briefly, three whole genome shotgun libraries were constructed containing inserts of approximately 3 kb, 8 kb, or 40 kb and sequenced to a depth of 9X using BigDye Terminators on ABI3730 sequencersb (Applied Biosystems). Shotgun reads were assembled with parallel PHRAP (http://www.phrap.org).

The MIT9215 genome was sequenced with a combination of approximately 20X coverage of 454 pysoquencing (454 Life Sciences) and standard Sanger sequencing of 3-kb insert libraries. All genomes were completed to finished quality with no gaps, except MIT9211, with one gap of less than 1 kb and an estimated error rate of less than 1 in 50,000 bases.

### Genome annotation.

We re-annotated 12 sequenced *Prochlorococcus* and four finished marine *Synechococcus* genomes by a uniform method for the purpose of this study. We used the gene prediction programs CRITICA [68] and GLIMMER [69]. The results from both programs were combined into a preliminary set of unique ORFs. Overlapping gene models from the two programs are considered the same gene if sharing the same stop position and in the same reading frame, in which case the gene start site of the CRITICA model is preferred. Coding genes that are shorter than 50 aa long are excluded unless they are conserved in more than one genome. Orthologous genes between two given genomes are assigned automatically using MicrobesOnline's [70] (http://www.microbesonline.org) genome annotation pipeline. The new annotations are also available at that site.

Two genes are considered orthologs if they are reciprocal best BLASTp hits and the alignment covers at least 75% of the length of each gene. An orthologous group includes all genes that are orthologous to any other gene in the group. The most common challenge of clustering orthologous genes is the risk of merging paralogous genes into one group. However, our method yields only 127 paralog-containing groups. In those cases, gene neighborhoods were also compared. Because a single missing ortholog effectively removes a gene from the core genome, the clusters that are absent in only one or two genomes were verified by BLAST search.

While the COG categories alone provide enough information to draw these conclusions about the membrane synthesis enzymes, there are some shortcomings. Some *Prochlorococcus* orthologous groups can be annotated with a gene name but not a COG (for example the LPS synthesis gene wcaK, or many photosystem genes like psbA), where literature searches show that they are likely involved in LPS synthesis. Other categories are hampered by the arrangement of the COG categories, which were not chosen with any particular focus on this system. For example, the category “Amino acid transport and metabolism” includes transporters and intracellular enzymes. When we found that transporters are among the most recently gained genes, we desired a way to group all of them by themselves. We decided the best approach was to group genes into five broad categories on the basis of keyword searches: membrane or cell wall synthesis, transporters, photosynthesis, DNA repair or modification, and other. HLI proteins were identified by their possession of six out of ten conserved residues in the motif AExxNGRxAMIGF, and lengths under 120 amino acids [32].

### Phylogenetic analysis.

16S rRNA and 16S-23S rRNA ITS region sequences were manually aligned in ARB and phylogenetic reconstruction using maximum parsimony, neighbor-joining, and maximum likelihood was done in PAUP [71]. Following the approach described in [33] to identify the phylogenetic relationship between the sequenced isolates, we aligned all core genes using clustalw using the protein sequence as reference. We randomly concatenated 100 alignments and constructed a phylogenetic tree using maximum parsimony and bootstrap resampled 100 times. The random concatenation was repeated 100 times and the average bootstrap values for concatenated alignments are reported in Figure 2. In addition, we also constructed a phylogenetic tree using maximum parsimony on each individual alignment and the most likely tree for each gene (plurality consensus tree based on 100 bootstraps) was identified. We also calculated the phylogenetic relationship based on the presence and absence of orthologous groups as previously described [34]. However, we used bootstrap instead of jack-knife resampling to test how well individual nodes were supported to ensure easy comparison with other phylogenetic trees.

Estimation of the timing of gene loss and gain events was as described using a maximum parsimony approach [35]. We used the phylogenetic tree in Figure 2C rooted between the *Prochlorococcus* and *Synechococcus* last common ancestors as a guide. We included the cost of a “gain” event in the tree's common ancestor node. We assigned a gene gain event twice the cost of a loss event, and in cases where two scenarios had equal scores we chose the one with fewer gains. We also tested a ratio of three to one, which changes the behavior of 117 genes.

### Metabolic reconstruction.

To predict the metabolic pathways present in the sequenced isolates, we ran Pathway Tools software [30] to generate a Pathway/Genome database (PGDB). This software creates gene, protein, reaction, small-molecule, and pathway objects based on Enzyme Commission (E.C.) numbers and enzyme names assigned in the genome annotation. We hand-curated the PGDB to eliminate unlikely pathways, and from it we created a pathway model of the central carbon metabolism [72]. To aid in the analysis of the core and flexible genes, we created a pseudogenome, Pan, which includes all genes from all isolates. We created another pseudogenome for the core genome. The database is available in flat file, BioPAX, and SBML format.

## Supporting Information

Figure S1 The Core Genome Includes Enzymes for Central Carbon Metabolism, Including the Calvin Cycle, Glycolysis, and an Incomplete TCA Cycle Producing Fumarate and 2-Oxoglutarate Some genomes, but not the core genome, also include *sdhAB*, encoding an enzyme for the reaction 1.3.99.1, the conversion of fumarate to succinate (Table 2). The pathway diagram includes the stuctures of intermediate metabolites, the locus name, in MED4, of the gene encoding each enzyme, the enzyme name, and the E.C. number.
(1.4 MB EPS)

Figure S2 The Core Genome Includes Enzymes for the Synthesis of All 20 Amino Acids The pathway diagram is annotated as in Figure S1.
(2.2 MB EPS)

Figure S3 The Core Genome Includes Enzymes for the Synthesis of Divinyl Chlorophyll The pathway diagram is annotated as in Figure S1.
(1.5 MB EPS)

Figure S4 The Core Genome Includes Enzymes for the Synthesis of the Cofactors NAD (A), Coenzyme A (B and C), and FAD (D) The pathway diagrams are annotated as in Figure S1. One reaction (2.7.1.33) in coenzyme A synthesis is highlighted; its enzyme (pantothenate kinase) has not been identified in the core or pan- genomes.
(1.3 MB EPS)

Figure S5 Islands of LL Genomes Not Represented in Figure 4
The dot plot shows the location of each gene, the ancestor in which it is estimated to be acquired, and when possible, the best match outside *Prochlorococcus*. The lower plot is the number of genes gained in a sliding window (size 10,000 bp, interval 1,000 bp) along the chromosome.
(4.2 MB EPS)

Figure S6 Islands of HL Genomes Not Represented in Figure 4
The dot plot shows the location of each gene, the ancestor in which it is estimated to be acquired, and when possible, the best match outside *Prochlorococcus*. The lower plot is the number of genes gained in a sliding window (size 10,000 bp, interval 1,000 bp) along the chromosome. When available, the locations of islands previously defined by hand are represented by shaded regions.
(6.1 MB EPS)

Table S1 All *Prochlorococcus* Orthologous Groups in This Study For each group, its locus names are given for those genomes in which it is found. Also given are the COG match [55], gene name, and description as assigned by MicrobesOnline (http://www.microbesonline.org).
(1.8 MB XLS)

Table S2 Prochlorococcus Core Genes Absent in Synechococcus 33 orthologous groups are shared by all *Prochlorococcus* but absent in some *Synechococcus*, and only 13 of those are absent in all *Synechococcus*. For each such orthologous group, its presence or absence in each of the four *Synechococcus* genomes in this analysis is given. Also given is the locus name for the gene in MED4, its COG match, and its gene name, if available.
(68 KB DOC)

Table S3 Genes Found in All Synechococcus but No Prochlorococcus The locus name for *Synechococcus* is given, in addition to the COG and gene name, if available.
(45 KB XLS)

Table S4 Genes Lost or Gained at Each Ancestor For each gene, the name and COG are given, in addition to a locus name. The role assigned is one of “nomatch,” “shortnomatch,” “conserved_unknown,” “hli,” “photosynthesis,” “DNA,” “membrane,” “transport,” or “other,” on the basis of keyword matches in the gene name, COG, or description. The latter five categories are reported individually in Figure 3B; the totals are reported in Figure 3A.
(1.5 MB XLS)

Table S5 The Most Common COGs in the Core and Flexible Genomes We used matches against the COG database as a first impression of the differences between the core and flexible genomes. The number of *Prochlorococcus* orthologous groups and the total number of genes in those groups, matching each COG is given. The top ten COGs matching the core and flexible genomes are shown.
(43 KB DOC)

Table S6 Orthologous Groups Found in All HL Isolates These include those exclusive to HL isolates and those shared with some, but not all, LL isolates, as indicated. Also given are the gene name, description, and COG assignments as in Table S1.
(114 KB XLS)

Table S7 Orthologous Groups Found in All LL Isolates As Table S6, but those found in all LL isolates.
(60 KB XLS)

Table S8 Notable Genes Exclusive to eMIT9313 Isolates These are orthologous groups from Table S1, each found only in MIT9303, MIT9313, and in some cases marine *Synechococcus*. This list includes only those genes with hypothetical functions and with no BLAST alignment against the other genomes. Note that some belong to COGs shared with other *Prochlorococcus* isolates, but their extreme sequence divergence suggests their precise roles differ.
(118 KB XLS)

## Abbreviations

HL: high-light
LGT: lateral gene transfer
LL: low-light
