# Supplementary material for: Patterns and Implications of Gene Gain and Loss in the Evolution of Prochlorococcus
Source: PLoS Genet. 2007 Dec 21;3(12):e231. doi: 10.1371/journal.pgen.0030231 (PMC2151091; doi:10.1371/journal.pgen.0030231)
Supplement: Supplementary file 1 [file pgen.0030231.st002.doc]

**Supplemental Table 2:** *Prochlorococcus* core genes absent in *Synechococcus*. 33orthologous groups are shared by all *Prochlorococcus* but absent in some *Synechococcus*, and only 13 of those are absent in all *Synechococcus*. For each such orthologous group, its presence or absence in each of the four *Synechococcus* genomes in this analysis is given. Also given is the locus name for the gene in MED4, its COG match, and its gene name, if available.

| **SynWH8102** | **SynCC9605** | **SynCC9902** | **SynCC9311** | **MED4 locus** | **COG** | **gene name** |
| --- | --- | --- | --- | --- | --- | --- |
| **Absent from *Synechococcus*** | | | | |  |  |
| 0 | 0 | 0 | 0 | PMED4_00681 |  |  |
| 0 | 0 | 0 | 0 | PMED4_02181 |  |  |
| 0 | 0 | 0 | 0 | PMED4_06781 | COG786:Na+/glutamate symporter [Amino acid transport and metabolism] | GltS |
| 0 | 0 | 0 | 0 | PMED4_06861 | COG1535:Isochorismate hydrolase [Secondary metabolites biosynthesis, transport, and catabolism] | EntB/pncA |
| 0 | 0 | 0 | 0 | PMED4_07061 |  |  |
| 0 | 0 | 0 | 0 | PMED4_08191 |  |  |
| 0 | 0 | 0 | 0 | PMED4_08941 | COG2146:Ferredoxin subunits of nitrite reductase and ring-hydroxylating dioxygenases [Inorganic ion transport and metabolism / General function prediction only] | NirD / hcaE |
| 0 | 0 | 0 | 0 | PMED4_11001 |  |  |
| 0 | 0 | 0 | 0 | PMED4_11581 |  |  |
| 0 | 0 | 0 | 0 | PMED4_11671 |  |  |
| 0 | 0 | 0 | 0 | PMED4_12731 |  |  |
| 0 | 0 | 0 | 0 | PMED4_15181 |  |  |
| 0 | 0 | 0 | 0 | PMED4_15741 |  | hli11 |
| **In some *Synechococcus*** | | | | |  |  |
| 1 | 0 | 0 | 1 | PMED4_00811 | COG2091:Phosphopantetheinyl transferase [Coenzyme metabolism] | Sfp |
| 0 | 1 | 1 | 1 | PMED4_02171 | COG492:Thioredoxin reductase [Posttranslational modification, protein turnover, chaperones] | TrxB |
| 0 | 0 | 0 | 1 | PMED4_02191 | COG3329:Predicted permease [General function prediction only] | sbtA |
| 1 | 1 | 0 | 0 | PMED4_02251 |  |  |
| 1 | 1 | 0 | 0 | PMED4_03481 |  |  |
| 0 | 0 | 0 | 1 | PMED4_03761 | COG5470:Uncharacterized conserved protein [Function unknown] |  |
| 1 | 1 | 1 | 0 | PMED4_05531 | COG1324:Uncharacterized protein involved in tolerance to divalent cations [Inorganic ion transport and metabolism] | cutA |
| 0 | 1 | 1 | 1 | PMED4_06771 |  | pcbA |
| 1 | 0 | 0 | 0 | PMED4_07161 |  |  |
| 0 | 0 | 1 | 0 | PMED4_07701 |  |  |
| 0 | 1 | 1 | 1 | PMED4_08901 | COG1528:Ferritin-like protein [Inorganic ion transport and metabolism] | / Ftn |
| 0 | 1 | 1 | 1 | PMED4_08921 | COG664:cAMP-binding proteins - catabolite gene activator and regulatory subunit of cAMP-dependent protein kinases [Signal transduction mechanisms] | Crp |
| 1 | 1 | 1 | 0 | PMED4_11181 |  |  |
| 0 | 1 | 1 | 1 | PMED4_11231 |  |  |
| 0 | 0 | 0 | 1 | PMED4_12131 |  |  |
| 1 | 1 | 0 | 1 | PMED4_12251 |  |  |
| 0 | 1 | 1 | 1 | PMED4_13361 | COG716:Flavodoxins [Energy production and conversion] | FldA |
| 0 | 1 | 0 | 1 | PMED4_14561 | COG63:Predicted sugar kinase [Carbohydrate transport and metabolism] / COG62:Uncharacterized conserved protein [Function unknown] |  |
| 1 | 1 | 1 | 0 | PMED4_15631 |  |  |
| 0 | 0 | 0 | 1 | PMED4_18811 |  |  |
