# Supplementary material for: Patterns and Implications of Gene Gain and Loss in the Evolution of Prochlorococcus
Source: PLoS Genet. 2007 Dec 21;3(12):e231. doi: 10.1371/journal.pgen.0030231 (PMC2151091; doi:10.1371/journal.pgen.0030231)
Supplement: Supplementary file 2 [file pgen.0030231.st005.doc]

**Supplemental Table 5:** The most common COGs in the core and flexible genomes. We used matches against the COG database as a first impression of the differences between the core and flexible genomes. The number of *Prochlorococcus* orthologous groups, and the total number of genes in those groups, matching each COG is given. The top 10 COGs matching the core and flexible genomes are shown.

| **COG Name** | **COG Description** | ***Prochlorococcus* Orthologous Groups** | **genes** |
| --- | --- | --- | --- |
| **Core** |  |  |  |
| COG524 | Sugar kinases, ribokinase family [Carbohydrate transport and metabolism] | 3 | 42 |
| COG456 | Acetyltransferases [General function prediction only] | 3 | 42 |
| COG1132 | ABC-type multidrug transport system, ATPase and permease components [Defense mechanisms] | 4 | 56 |
| COG740 | Protease subunit of ATP-dependent Clp proteases [Posttranslational modification, protein turnover, chaperones / Intracellular trafficking and secretion] | 4 | 56 |
| COG745 | Response regulators consisting of a CheY-like receiver domain and a winged-helix DNA-binding domain [Signal transduction mechanisms / Transcription] | 4 | 56 |
| COG596 | Predicted hydrolases or acyltransferases (alpha/beta hydrolase superfamily) [General function prediction only] | 4 | 56 |
| COG1028 | Dehydrogenases with different specificities (related to short-chain alcohol dehydrogenases) [Secondary metabolites biosynthesis, transport, and catabolism / General function prediction only] | 4 | 56 |
| COG465 | ATP-dependent Zn proteases [Posttranslational modification, protein turnover, chaperones] | 4 | 56 |
| COG568 | DNA-directed RNA polymerase, sigma subunit (sigma70/sigma32) [Transcription] | 5 | 70 |
| COG451 | Nucleoside-diphosphate-sugar epimerases [Cell envelope biogenesis, outer membrane / Carbohydrate transport and metabolism] | 6 | 112 |
|  |  |  |  |
| **Flexible** |  |  |  |
| COG457 | FOG: TPR repeat [General function prediction only] | 7 | 8 |
| COG1028 | Dehydrogenases with different specificities (related to short-chain alcohol dehydrogenases) [Secondary metabolites biosynthesis, transport, and catabolism / General function prediction only] | 7 | 20 |
| COG2274 | ABC-type bacteriocin/lantibiotic exporters, contain an N-terminal double-glycine peptidase domain [Defense mechanisms] | 7 | 20 |
| COG463 | Glycosyltransferases involved in cell wall biogenesis [Cell envelope biogenesis, outer membrane] | 8 | 25 |
| COG1943 | Transposase and inactivated derivatives [DNA replication, recombination, and repair] | 10 | 11 |
| COG399 | Predicted pyridoxal phosphate-dependent enzyme apparently involved in regulation of cell wall biogenesis [Cell envelope biogenesis, outer membrane] | 11 | 16 |
| COG5010 | Flp pilus assembly protein TadD, contains TPR repeats [Intracellular trafficking and secretion] | 12 | 20 |
| COG451 | Nucleoside-diphosphate-sugar epimerases [Cell envelope biogenesis, outer membrane / Carbohydrate transport and metabolism] | 13 | 33 |
| COG3063 | Tfp pilus assembly protein PilF [Cell motility and secretion / Intracellular trafficking and secretion] | 17 | 24 |
| COG438 | Glycosyltransferase [Cell envelope biogenesis, outer membrane] | 25 | 89 |
